# Correspondence

**Published:** 1874-11

**Authors:** E. T. Dorland

**Affiliations:** Buffalo


### Correspondence.

*Editors Buffalo Medical Journal:*

The following case being one of rather unfrequent occurrence, I thought it might be of sufficient interest to report, and the success of the treatment may tend to allay the fears of some practitioner in the future of resorting to the same radical treatment, under similar circumstances. The case was one of the most extensive hemorrhoidal tumors I ever saw, heard or read of. The subject, Miss Barmore, was a patient of mine years ago when practicing in Dutchess county. For years she was under treatment for bronchitis, female difficulties, and general debility. About twelve years ago she commenced having attacks of hemorrhage from the bowels, which gradually increased till so severe, at times, that her life seemed in imminent peril. About this time much difficulty began to be experienced in procuring evacuations of the bowels, and soon after this there was discovered tumors, protruding after each dejection, which were with difficulty returned into the rectum. These tumors continued to increase in size, and the hemorrhage as frequent and profuse as before their appearance. Extirpation of the tumors was proposed from time to time, but the exceedingly delicate state of the patient's health, especially the diseased condition the lungs appeared now to be taking on, seemed in my judgment and in the opinion of others who were consulted, to render the propriety of an operation extremely doubtful. So the case was in the main left to nature, physicians and friends supposing the patient must succumb to the combination of difficulties afflicting her. So in this miserable condition she dragged along for years, her bowels only moving after long continued efforts, aided by copious injections, and followed almost invariably by the loss of from an ounce or two to half a pint of blood. A great many times she lost more than a pint at a single evacuation. The aggregate amount of blood lost in her case, if accurately known, would, no doubt, seem incredible. I could not have believed their statements possible, had I not been many times cognizant of the facts as to the amount of blood lost, by personal observations. During these years the tumors continued to grow until they attained an enormous development, the mass being about the size of a teacup, when fully protruded.

About three years ago, notwithstanding this long existing and still profuse sanguineous discharge, there appeared evidences of a marked improvement in her general health. A general dropsical condition however existed, and continued to the time of the operation. On being advised from time to time, by correspondence of her gradually returning health, I concluded it might be reasonably safe to operate for the removal of the immense growth from her rectum. I consulted with Dr. Lynde of this city, and he advised an operation. The patient, on being informed of our opinion, decided to have it done at once, as her condition was so utterly miserable. Having a *childish* desire to be under the care of her old physician, she determined to come to Buffalo to have the operation performed. She accordingly came and was operated on the fourteenth day of May last, Dr. Lynde performing the operation, assisted by myself. The tumors were brought as far external as possible, and the whole mass ligated; the tumors being very large and extensive at their base, required transfixing at several points; No less than four double ligatures and four or five single ones were used in ligating the mass. Contrary to expectation very little inflammatory action followed. The mass sloughed off nicely within two weeks, and the parts readily healed. A rapid improvement in the general health followed—no hemorrhage from the bowels since, the dropsical condition disappearing, and the most gratifying of all to the patient, the bowels are moving easily, promptly, and regularly, unaided by medicines or enemas. On the whole, a very satisfactory result to both patient and physicians.

E. T. DORLAND.

BUFFALO, Sept. 9, 1874.

